# Hope Aspects of the Women’s Experience after Confirmation of a High-Risk Pregnancy Condition: A Systematic Scoping Review

**DOI:** 10.3390/healthcare10122477

**Published:** 2022-12-08

**Authors:** Mónica Antunes, Clara Roquette Viana, Zaida Charepe

**Affiliations:** Center for Interdisciplinary Research in Health, Institute of Health Sciences, Universidade Católica Portuguesa, 1649-023 Lisbon, Portugal

**Keywords:** high-risk pregnancy, hope, life experience, mental health, pregnancy complications, prenatal diagnosis, review

## Abstract

Background: Pregnancy is a period of transformation, hope, expectation, and worry for women and their families. A high-risk pregnancy refers to a pregnancy in which the mother and/or fetus are at greater-than-normal risk of complications, and it evokes a range of emotional and psychological experiences that largely depend on the care and support provided by health professionals. The purpose of this review is to summarize the existing literature on the lived experience of hope in women facing a high-risk pregnancy related to their own health and/or medical conditions related to the fetus. Methods: This review followed the Joanna Briggs Institute’s methodology. No limits on a date were applied to the search. Identified titles and abstracts were screened to select original reports and were cross-checked for any overlap of cases. We included studies that emphasized the experience of hope of pregnant women dealing with a pregnancy complication. Main Results: According to the results of the present scoping review, we found two main dimensions: women experiencing a high-risk pregnancy themselves and prenatal diagnosis. In both cases, the women were in a dilemma between hope and hopelessness. Conclusion: The findings demonstrate that women facing high-risk pregnancies struggle with multiple fears and concerns about their own health and the fetus’s health. Further research is needed to identify best practices for the care provided to the vulnerable populations.

## 1. Introduction

In 2017, approximately 810 women died from preventable causes related to pregnancy and childbirth [1]. Globally, more than 20 million women are at risk of high-risk pregnancies, which results in an estimated 830 deaths per day, more than 99% occurring in developing countries, and is more frequent among rural women and adolescents [2]. Nearly 22% of pregnant women develop a high-risk pregnancy [3]. As part of the sustainable development goals (SDG), countries have agreed on a new target to accelerate the decline of maternal mortality by 2030. The World Health Organization (WHO) considers high-risk pregnancies a major public health challenge, addressing the healthcare needs as a priority [1], and includes an ambitious target: decreasing the global maternal mortality rate to less than 70 per 100,000 births, with no country having a maternal mortality rate of more than twice that of the global average [1]. 

In most cases, the birth of a child is an experience filled with joy and happiness. However, when a mother is diagnosed with a medical condition or the child with a congenital anomaly, the parents’ experience can take on a different meaning [4]. A high-risk pregnancy is defined as any pregnancy in which there is a medical factor, maternal or fetal, that may potentially adversely affect the outcome of the pregnancy [5]. The most common maternal complications are gestational diabetes, preeclampsia and eclampsia, depression, sexually transmitted diseases, preterm labor, and placenta previa [3], while the most frequent congenital anomalies are heart malformations, neural tube defects, and Down syndrome. An anomaly may be genetic, infectious, or environmental in origin. In most cases, however, the cause is unknown, which makes it more difficult for parents to understand and accept the situation [6]. In many countries, congenital anomalies are important causes of perinatal morbidity and mortality, which can lead to chronic disabilities which may have severe consequences on individuals, families, healthcare systems, and societies [7]. The sudden sense of grief, loss, and guilt coupled with a fear of the unknown future often produces a great deal of anguish for parents [5], especially in pregnant women.

Every pregnancy is unique, physiologically, and a natural episode in a woman’s life, and all pregnant women experience physical, mental, and social changes in different manners [3]. The ways in which the changes inherent to this transition moment are integrated and experienced seem to be directly related to the woman’s personality, marital status, family, and social support [8]. 

Under normal conditions, pregnancy is a natural transition for women and their families. When a pregnant woman is diagnosed as being high-risk, she may find it difficult to cope with this new reality, leading to psychological and emotional consequences such as fear, guilt, shock, grief, frustration, worry, loneliness, and isolation [9]. A high-risk pregnancy with complications is one of the risk factors causing pregnant women to experience psychosomatic problems such as anxiety, depression, and distress, and to suffer impairments in their health [10]. A qualitative study also showed that besides medical problems, women experience behavioral, affective, and emotional problems. Moreover, they are also at risk of sociocultural and financial strains that often lead to feelings such as uncertainty, concern, and insecurity [11]. 

Women’s coping strategies during pregnancy demand multiple challenges in which hope and resilience play essential roles in managing stress and mental health. Hope is defined as the perceived ability to find pathways to desired goals and to motivate oneself to use those pathways [12]. As a focus of nursing practice, hope is defined as “(…) feelings of having possibilities, trust in others and in future, zest for life, expression of reasons and will to live, inner peace, optimism, associated with setting goals and mobilization of energy” [13] (p. 1). In a study published in 2019, the author defends that helping individuals and their family members to find meaning in suffering and striving to invoke a sense of constructive hope should be a fundamental aspect of health care [14]. Inspiring appropriate hope might be considered a concern to healthcare professionals whatever their specialty [14]. In this review, the lived experiences of women’s hope comprises spheres and dimensions [14]. According to Dufault and Martocchio, there are six dimensions associated with the concept of hope [15]: affiliative (relationships with women and God that can be expressed by individuals who seek or are receptive to others’ help in hope); affective (sensations and emotions that are part of the process of hope); cognitive and behavioral (interpret and judge in relation to hope and actions orientation towards the desired outcomes, respectively); contextual (life situations that surround, influence, and are a part of women’s hope); and temporal (focuses upon hoping within the women’s experience of time). 

Therefore, the aim of this review was to assess the state of knowledge regarding the lived experience of hope among women facing high-risk pregnancies that may endanger the health of the mother and/or fetus. This openness to the possibility of working hope in a perspective other than cure, and the commitment of nurses in the practice of promoting hope as a duty of care and a standard of good clinical practice, has led to the need to investigate the concept and look for new ways to better-inspire hope in women who face pregnancies with complications in the context of their health and/or that endanger the health of the fetus.

## 2. Materials and Methods

The protocol and proposed systematic review were drawn and conducted in accordance with the JBI methodology for systematic reviews [16]: define search strategy, study selection, assessment of methodological quality, data extraction, and data synthesis. The protocol was registered prospectively with the Open Science Framework on 26 May 2022 (https://osf.io/u9ns8 (accessed on 12 September 2022)). Registration DOI: 10.17605/OSF.IO/U9NS8.

### 2.1. Review Questions

The following question guided this scoping review: What are the hope aspects related to the women’s lived experience of a pregnancy that continues after confirmation of a high-risk pregnancy diagnosis? This question was divided into the following sub-questions: What type of evidence or study design exists in the area of women’s life experience of pregnancy research in relation to hope aspects after confirmation of a high-risk pregnancy diagnosis?; What aspects of hope in the life experience of pregnant women have been addressed during health care associated with a high-risk pregnancy?; What are the gaps in the nursing research in relation to hope interventions in the context of care for women with high-risk pregnancies? 

### 2.2. Inclusion Criteria

#### 2.2.1. Participants

Both the exploratory and textual components of this review considered qualitative and quantitative studies that include women’s lived experiences of pregnancy with a high-risk condition that may affect their own health or their baby’s health, including the prenatal diagnosis of a congenital anomaly, regardless of race, nationality, level of education, or religious affiliation. The study could also include any patient with an age above 18, primigravida and multigravida, and in the second or third trimester of their pregnancy. This review excluded any studies focusing on healthcare professionals and pregnant women who were healthy and did not have any risk factors for high-risk pregnancies.

#### 2.2.2. Concept

This review considered studies that explore woman’s hopeful experiences when proceeding with a high-risk pregnancy diagnosis, which may have included maternal and/or fetal complications. The included studies may or may not have directly addressed hope in their proposals having, however, addressed hope-related experiences and related concepts in their findings or women’s expectations. The aspects related to women’s experiences of hope included psychological well-being; uncertainty; finding a sense of normality; finding a new meaning to life experiences; setting realistic goals and objectives; imagining possibilities and seeking alternative solutions; an ability to share their pregnancies with other women and/or health care professionals; and expressing positive personal transformation and identifying positive psychological factors. The aspects related to women’s expectations will include difficulties in thinking about the child’s future; condition-related expectations (positive or negative outlook); and concerns about acceptance by family and social networks.

#### 2.2.3. Context

Women with high-risk pregnancies are usually referred to larger health centers for better treatment. In this review, women were assisted in high-risk maternal–fetal health consultations, which included the literature from any country or sociocultural setting.

#### 2.2.4. Types of Sources

This review considered quantitative, qualitative, and mixed study designs for inclusion. Systematic reviews and meta-analyses were also considered in this review.

### 2.3. Search Strategy 

The search strategy aimed to find studies published in the Portuguese, English, or Spanish languages, with no date limit. As recommended in all JBI types of reviews, a three-step search strategy was carried out [16].

An initial limited search took place on the EBSCO platform, where the databases CINAHL and MEDLINE (PubMed) were selected, as well as the Google Scholar platform. The search was performed using the keywords included in the PCC question. A second search with all identified keywords and index terms was undertaken across all of the included databases, using Boolean descriptors such as “OR” and “AND”. A search with all identified keywords and index terms was used to develop a full search strategy for PubMed (see Appendix A). In the third stage, reference lists of sources selected from the full text and/or included in the review were examined. The databases searched include CINAHL Complete (from EBSCO); Pubmed; Nursing and Allied Health Collection (from EBSCO); PsycINFO; Mediclatina (from EBSCO); and Scopus. 

### 2.4. Study/Source of Evidence Selection

Following the search, all identified citations were compiled and uploaded to Mendeley version 2.71.0 (Mendeley Ltd., Elsevier, Amsterdam, Netherlands), and duplicates were removed. After a pilot test, the titles and abstracts were screened by two or more independent reviewers for assessment against the inclusion criteria for the review. The resulting reference list was then uploaded to Rayyan (Qatar Computing Research Institute, Doha, Qatar). In the second phase of screening, the references of the selected studies were reviewed, and relevant studies were identified for a full review. The studies were classified into one of three categories: included, excluded, and uncertain. Later, the full texts of the “included” and “uncertain” studies were retrieved to identify potentially relevant studies, and their full citation details were imported into Rayyan.

The full texts of the selected citations were assessed in detail according to the inclusion criteria by two independent reviewers. Reasons for the exclusion of sources of evidence in full text that did not meet the inclusion criteria were recorded and reported in the scoping review. Any disagreements that arose between the reviewers at each stage of the selection process were resolved through discussion, or with an additional reviewer/s. The results of the search and the study-inclusion process are reported and presented in a preferred reporting items for systematic reviews and meta-analyses extension for scoping review (PRISMA-ScR) flow diagram [17]. 

### 2.5. Assessment of Methodological Quality 

In describing the quality of the selected articles (*n* = 15), the studies were appraised by all of the authors. Divergent views regarding the critical appraisal were reviewed until a consensus was achieved. The Hawker et al. [16] assessment tool, with a four-grade scale (1 = very poor; 2 = poor; 3 = fair; 4 = good) was used. The total scores ranged between 9 and 36, and higher scores indicated a higher quality. An article’s quality appraisal was centered on the following items: 1—abstract and title; 2—introduction and aims; 3—method and data; 4—sampling; 5—data analysis; 6—ethics and bias; 7—results; 8—transferability or generalizability; and 9—implications and usefulness.

### 2.6. Data Extraction

**Figure 1:**
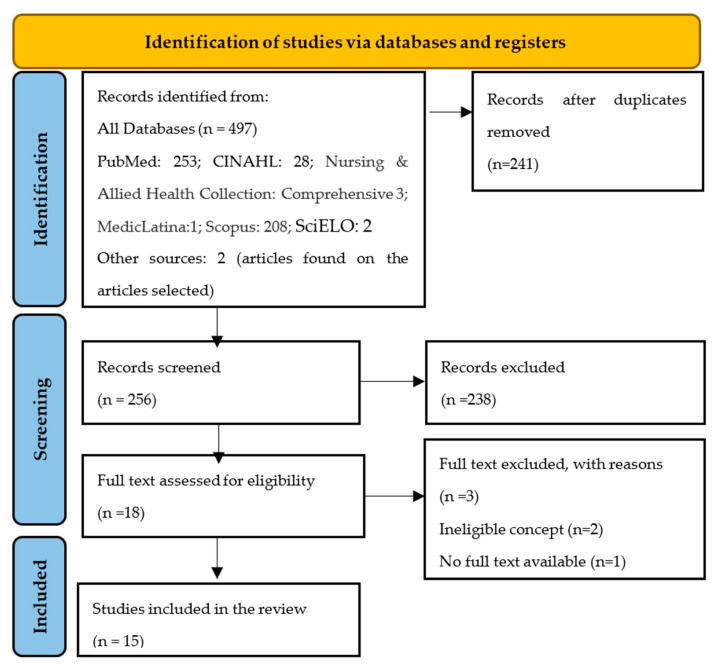
Flowchart of the selection and screening process of the systematic review articles according to the PRISMA method.

After the analyses of the title and abstract, duplicates and articles that did not correspond to the topic were excluded. Primary studies published in Portuguese, English, and Spanish were included, with no time limit. The search was conducted on 16 March 2022, with an update on 16 September 2022. A total of 479 articles were excluded, and another 4 full-text articles were excluded, two of them with an unsuitable concept and for the other two we did not receive a response from the authors to access the full text, leaving 15 for analysis (Figure 1). The titles and abstracts identified during the search were independently reviewed by the authors using the inclusion and exclusion criteria. The decision of whether to include or exclude studies was made by mutual agreement. 

### 2.7. Data Synthesis 

The 15 studies eligible for SR are described in Appendix B. The results are presented in narrative form. Considering the JBI guidelines [16], the synthesis of relevant data collected from each article was composed of the following elements: the identification of the article, hope and hopelessness experiences or expectations, aims, study design, study population/sample, context, population characteristics, typology, and main results.

## 3. Results

The generated demand resulted in 495 titles. After applying the inclusion/exclusion criteria and excluding duplicate studies, 15 studies were eligible. Of these, 14 were in the English language and 1 was in the Portuguese language (Brazil).

### 3.1. Characteristics of Sources of Evidence

The main characteristics of the fifteen articles were as follows: fifteen primary studies, four of which were conducted in Iran, two in the United States of America, one in Brazil, one in the United Kingdom, one in Belgium and the United Kingdom, one in Sweden, one in Australia, one in Paris, one in Africa, one in Malaysia, and one in Thailand. Only one of the studies found was quantitative (descriptive, prospective, and longitudinal); the rest of the studies were qualitative.

The selected studies were published between 2004 and 2021. The ages of the women ranged from 18 to 45 years. The sample sizes varied from nine to seventy-two women who used the services of specialized centers, hospitals, or clinics for the specific medical condition presented.

There are studies (see Appendix B) that have focused on women’s experiences with maternal medical conditions [18,19,20,21,22,23,24,25,26,27], and other studies that have focused on the prenatal diagnosis of fetal pathology [28,29,30,31,32]. 

Few studies have addressed the aspects of hope during health care associated with high-risk pregnancies, and only one study was quantitative, while fourteen studies were qualitative.

The empirical studies described the lived experiences of women during pregnancy in the second and third trimesters with varied medical conditions. 

Authors such as Behboodi-Moghadam, Khalajinia, Nasrabadi, Mohraz and Gharacheh (2016), and Sanders (2008) explored the experiences of women diagnosed with HIV at each stage of pregnancy [18,20,23]. This same medical condition was the focus of other studies that examined these experiences in the prenatal period in South Africa [25], Thailand [24], and Brazil [27]. On the other hand, authors such as Tong, Brown, Winkelmayer, and Craig and Jesudason described the beliefs, values, and experiences of pregnancy in Australian women with CKD to inform on pre-pregnancy counseling and pregnancy care [21].

The experiences and perceptions of women with high-risk pregnancies are focused on topics regarding health and care practice issues/needs [22,30]. Tayeh, Jouannic, Mansour, and Kesrouani and Attieh explored patients’ perceptions of the prenatal diagnosis of fetal cardiac pathology and their reasons for deciding to continue the pregnancy despite being eligible for the medical termination of their pregnancy [30]. Norhayati, Hazlina, Hussain, Asrenee and Sulaiman examined women’s experiences of near-misses and their perceptions of quality of care in a retrospective study [26].

A theoretical framework for the process of adaptation following a fetal anomaly diagnosis was provided based on the proposal in the study of Lalor, Begley and Galavan [28]. In this study, data was collected from Irish women’s experiences of carrying a baby with a fetal abnormality to the end and beyond birth [28,29]. Integrated into the fetal abnormality, other authors describe the women’s reactions to the discovery of fetal hydronephrosis in the context of uncertainty about prognosis [19] and the women’s experiences during pregnancy with a child with a known, nonlethal congenital abnormality [32]. 

The concepts and strategies that women in the UK and Belgium use when considering maternal–fetal surgery as an option for the management of spina bifida in their fetus, and how this determines the acceptability of the intervention, were researched in [31].

### 3.2. Critical Appraisal within Sources of Evidence 

The study’s quality was high. Methods, ethics, and bias, as well as transferability, were the studies’ main limitations, and the critical appraisals ranged from 32 to 36 (see Appendix C).

### 3.3. Results of Sources of Evidence

According to the results of the present systematic review, women who experience a high-risk pregnancy or a prenatal diagnosis of fetal abnormality found themselves in a dilemma between hope and hopelessness and, in some cases, a second dilemma between terminating or continuing the pregnancy. 

To answer the question: “What are the hope aspects related to the women’s life experience of a pregnancy that continues after confirmation of a high-risk pregnancy diagnosis?”, we were able to find two subcategories within the main category of “Hope experiences in women with a high-risk pregnancy.” We can distinguish hopelessness and hope experiences. In nine studies it was possible to analyze that the risk involved was related to the women themselves in the context of their medical conditions (see Table 1). 

The studies we found related well to the hope and hopelessness women experience in the context of prenatal diagnoses that could endanger the fetus’s health (see Table 2). 

Some studies have established a link between women’s experiences of hope and the positive outcomes associated with this perception. The following results in women with medical conditions include the change from their experiences of uncertainty to new solutions and/or possibilities about a future uncertainty. These women focus their hope on the child and the privilege of experiencing pregnancy. 

On the other hand, studies conducted with women with prenatal diagnoses are focused on outcomes that are associated with a positive future for the child and the realization that they made the right decision when they decided not to terminate the pregnancy. 

The hope experiences related to women have more expression in the group of women who have the prenatal diagnosis without medical conditions in themselves. However, after a prenatal diagnosis of a fetal anomaly in the initial phase, some women hoped that they would hear that the diagnosis was a mistake [28]. 

Each person has different coping strategies when faced with stress [19]. Women should be clearly informed about the options and decisions they may need to make in cases of abnormal tests and prenatal screenings [29,32].

## 4. Discussion

In the study of high-risk pregnancies with different medical problems, hopelessness experiences are implicitly related to pregnancy worries, concerns about the child, future pregnancy, relationships and support with others, and higher costs [18]. 

When the risk was associated with the diagnosis of HIV during pregnancy, the attribute that emerged in all studies was fear of the cruelty of stigma, stereotyping, discrimination, and judgment [20,23,24,25,27]. 

Studies also showed the existence of concerns about the transmission of the virus to the baby and possible effects on their health [20]; emotional distress, ambivalence about pregnancy and motherhood [23], an association with negative self-image, loneliness, feelings of isolation, fears of loved ones, blame, and that they avoided any romantic relationships, and fears of being sick, going to the hospital, or dying [25]. In the same population, Ross et al. found that women perceived their lives as a struggle [24], such as with shock, fear, anxiety, and depression; with sharing one’s struggling with others and that they struggle to care for their baby, especially after birth; and that they struggle through ups and downs. The consciousness of fragility, noxious self, denied motherhood, social jealousy, and fear of genetic transmission have been described in the study of women with chronic kidney disease [21]. 

Studies focused on maternal near-miss experiences showed many fears and concerns [22,26]: fear of being unable to become pregnant again, fear of raising their child without siblings, fear of carrying about their child without a mother, fear of remarriage of a spouse if the spouse wanted more children, fear of becoming pregnant again and experiencing postpartum complications, fear of not being able to adjust to the complications and grieving for a long time, and guilt, intolerance of pain, irritability, and postpartum depression.

In the optical of mental health, other emotions have an expression in this analysis, such as anxiety, discouragement, and numbness, fear for their own life and the life of their baby, feelings of death, and feelings of an incomplete self because they are a woman without a uterus and a baby [26].

In a study that looked at the experiences of women with various pathologies during pregnancy, hope was found to be related to the adaptation to challenges, the belief that conditions will improve, and being hopeful about the future [18]. 

Children were seen as a “divine gift”, a chance to correct past mistakes, and to be good, loving mothers [20,23,27]. 

Religiousness resurfaced with the wish for a child to give meaning to their life’s and to help with the construction of a female identity, because to be a mother was stronger than any problem [27]. Hope was also identified in protecting children from contracting the disease and from stigma in the case of HIV disease [23,24,25].

In general, the studies reported that a positive pregnancy experiences was an important source of support and hope for women [27]. By participating in spiritual practices, women believed that God would respond to their needs and take care of their children [20,25,27]. Spirituality and resorting to God and Imams are the most common attributes of hope found in the found studies [18,22,25,26,27].

Other forms of adaptations included the natural maternal disposition to focus on their child, which was a motivator to seek treatment and a source of strength to continue living [21,26].

When a prenatal diagnosis occurs, studies identified the following women’s hopelessness attributes: the sense of disbelief, stress, doubting the struggle, shock, anger, and fear of developing a bond with the baby who may die [19,28,29,32]; anxiety and fears about the unknown [19]; guilt by the loss of the perfect baby [32].

Only one study analyzed the hopeless experience in the case of a woman who had maternal–fetal surgery [31]. Emotions such as uncertainty about their future child’s quality of life, fear of potential complications of the surgery and the possibility of losing their unborn child, fear of not waking up after the operation, and post-traumatic stress and depression were related [31].

In the rebuilding phase after prenatal diagnosis, a positive vision of the future seems to develop (whatever that may be) as the woman processes her experiences in such a way as to reconstruct the future and adjust her earlier beliefs about pregnancy and the world in general [28]. For example, in the context of fetal surgery, women felt strong feelings of responsibility and determination to do anything to improve their future child’s health outcomes [31].

The study conducted by Oscarsson et al. found that women’s experiences of hope were based on going through a crisis and knowing that they were doing the right thing [19]. Irani et al. compared the emotional experiences of women that decided to continue or terminate the pregnancy after the prenatal diagnosis of fetal anomalies [29]. The results identified in this population include a dilemma between hope and worry maintained by a positive attitude toward childbirth to cope with the situation and/or a return to normality [29]. In another study that compared the experiences between continuing and terminating the pregnancy, the authors cited religious beliefs and convictions and the belief that the baby would survive after birth [30]. Other sources of hope were found when there were good sources of information, time to prepare, support from family and friends, spiritual beliefs, staying busy with work and other activities and empathizing with the baby [32].

## 5. Conclusions and Implications for Practice

This review demonstrated the meanings of the lived experiences of women with high-risk pregnancies due to maternal medical conditions or prenatal diagnoses. 

The hope aspects related to the women’s lived experiences of pregnancies that continued after the confirmation of a high-risk pregnancy diagnosis were addressed during health care and were analyzed with a personal woman’s attributes.

The type of evidence or study design that exists in women’s life experiences of pregnancy research in relation to hope aspects after the confirmation of a high-risk pregnancy diagnosis is mostly qualitative. In the studies analyzed, the qualitative design stood out in terms of methodology (14 of the 15 studies analyzed in this review). This aspect is related to the type of the main review question, as we intended to understand the experiences of hope and not measure them quantitatively.

In the studies that explored high-risk pregnancies, the main issues identified were women’s fears for their own lives and the lives of their babies, the impossibility of getting pregnant again, postpartum complications, and an incomplete self. On the other hand, women facing a prenatal diagnosis of fetal anomalies faced the fear of developing an attachment to a baby who may die, the loss of the perfect baby, and the fear of possible complications of treatment. In both cases, women went through phases with feelings of emotional distress, ambivalence, disbelief, stress, struggle, anxiety, and depression.

Most women facing a high-risk pregnancy used religion, spirituality, and faith in God as coping mechanisms. Some others relied on good sources of information, time to prepare, support from family and friends, employment at work, and empathy with the baby. These women had a strong sense of responsibility for the treatment and saw it as an opportunity that they had been given. If they decided to continue with the pregnancy, they focused on the idea of doing the right thing. They hoped for the best possible outcome and that everything would be okay in the end.

In relation to hope interventions in the context of care for women with high-risk pregnancies, the gaps in the nursing research are about hope interventions. The results of different studies describe an implicit proposal to develop research about hope interventions in the context of care for women with high-risk pregnancies. However, we believe that women who are well informed about their situation and treatment are most likely to adapt and comply with treatment. In addition, recognizing the benefits of religious faith in situations of uncertainty is important and helps women to adjust to challenging situations. 

It is extremely important to monitor women’s emotional and psychological reactions after a prenatal anomaly diagnosis, not only throughout the pregnancy but also in the postnatal stages. Nurses and midwives have a privileged position in relation to women and can help them overcome difficult challenges in the present and future.

To establish a comparison between women’s responses in the different conditions (maternal medical situations and prenatal diagnoses), it is highly important to increase our understanding of the impacts of these experiences in this population.

## 6. Limitations 

The present scoping review was based mainly on qualitative studies conducted on a limited number of mothers with high-risk pregnancies, so the results cannot be generalized to similar populations. 

## Figures and Tables

**Table 1:** Hopelessness and hope experiences in women with medical conditions.

Hopelessness Experiences	Hope Experiences
- Worries and concerns about the child, future pregnancy, relationships, and higher costs [18] - Stigma, stereotyping, discrimination, and judgment [20,23,24,25,27] - Emotional distress and ambivalence [23] - Negative self-image, loneliness, feelings of isolation, and blame [25] - Fears for their own life and the life of their baby, unable to become pregnant again, and postpartum complications [26] - Struggle, shock, anxiety, and depression [24] - Conscious of fragility and fear of genetic transmission [21] - Not being able to adjust to the complications, grieving for a long time, guilt, intolerance to pain, and irritability [22,26]	- Adaptation to challenges [18] - Belief that conditions will improve [18,27] - Positive about the future [18,25,28] - Children as a “divine gift”, a chance to correct past mistakes, to be good, a loving mother [20,23] - Children as a meaning to their life and a motivator that helps to face challenges [24,27,28] - Pregnancy as construction of the female identity [27] - Medicines as hope, protecting their own life and protecting an unborn child [24,25] - Positive pregnancy experiences as a source of hope [21,24] - Religion, spirituality, faith in God [18,20,22,26,27] - Valuing life, gratitude, and focus on what is good [21]

**Table 2:** Hopelessness and hope experiences in women with prenatal diagnoses.

Hopelessness Experiences	Hope Experiences
- Disbelief, stress, and struggle [28] - Fear of developing a bond with a baby who may die [28] - Anxiety and fears about the unknown, worry, stress, and depression [19,28,29,32] - Feelings of grief, shock, anger, panic, distress, and guilt [29,32] - Loss of perfect baby and fear of potential complications of the treatment [31]	- Positive vision of the future [28,31] - Reconstruct the future and adjust earlier beliefs [28,31] - Strong feelings of responsibility about the treatment, seen as an opportunity that was given [31] - Determination to do anything that would improve their future child’s health outcomes [19] - The sense of doing the right thing by continuing the pregnancy [29] - Hope for the best possible outcome and that everything would be all right at the end [29] - Positive attitude toward childbirth [29,30,31] - Hope to return to normal [30] - Other sources of hope, such as religious and spiritual beliefs, good sources of information, time to prepare, support from family and friends, staying busy at work, and empathizing with the baby [30]

## Data Availability

Not applicable.

## Appendix A

**Table:**

Scheme	Pubmed Search Equation	Results
#1	pregnant women OR maternal OR expectant mother	482,511
#2	high-risk pregnancy OR pregnancy complications OR obstetric complications OR medical condition OR obstetric health care	2,526,150
#3	hope OR hopelessness	89,314
#4	Qualitative Studies OR qualitative research OR phenomenological research OR Experiences OR Perceptions OR Attitudes OR Feelings OR meaning OR need	3,843,920
#5	1 AND 2 AND 3 AND 4	253

## Appendix B

**Table:**

Author(s) Year Publication Country	Hope and Hopeless Experiences or/and Expectations	Aims	Study Design	Study Population/Sample	Context	Population Characteristics and Typology	Main Results
The lived experience of women with a high-risk pregnancy: A phenomenology investigation Badakhsh, Hastings-Tolsma, Firouzkohi, Amirshahi & Hashemi (2020) Iran [18]	Hopelessness experiences are implicit with pregnancy concerns, worries about the child, future pregnancy, relations and support with others and increase cost Hope is implicit on adaptation to the challenges, believing that the condition would improve and being hopeful about the future. Spirituality, resorting to the God and Imans.	To describe the lived experience of women during HRP	Phenomenological study	Pregnant women	Public health centre in a large urban city in south-east Iran	- Primigravida or multigravida - Second or third trimester of pregnancy - varied high-risk medical conditions - varied age and levels of education - able to speak Persian language	Four thematic categories were extracted: - Challenges of family in HRP - Challenges for future pregnancies - Challenge of anticipation for motherhood (involving others, increased costs, treatment, and life management) - Challenge of adaptation
Recasting Hope: A process of adaptation following fetal anomaly diagnosis Lalor, Begley & Galavan (2009) Ireland (United Kingdom) [28]	Hopelessness implicit on sense of incredulity, stress, the dually of the fight. Fear of developing a bond with the baby that could die. Initially some women hoped they would hear that the initial diagnosis was a mistake. Finally, in the rebuilding phase it would appear that the emergence of a positive vision of the future (whatever that might be), as the woman works through her experience in ways that permit her to reconstruct the future and adapt her previously held beliefs about pregnancy in particular and the world in general.	To provide a theorical framework of the process of adaptation following fetal anomaly diagnosis based on women’s experiences of carrying a baby with a fetal abnormality up to the end and beyond the birth	Grounded theory study	Pregnant women carrying a baby with fetal abnormality	Fetal medicine unit of a major Dublin maternity hospital	All Irish women. Forty-one women, eleven primigravidae and 30 multigravidae. Thirthy-one women continued the pregnancy and ten travelled to UK to access termination of pregnancy services not available within the state. Forty women were married or partnered and although not all pregnancies were planned, all were wanted.	Recasting Hope, the process of adaptation following diagnosis is represented temporally as four phases: ‘Assume Normal’, ‘Shock’, ‘Gaining Meaning’ and ‘Rebuilding’. Some mothers expressed a sense of incredulity when informed of the anomaly and the ‘Assume Normal’ phase provides an improved understanding as to why women remain unprepared for an adverse diagnosis. Transition to phase 2, ‘Shock,’ is characterised by receiving the diagnosis and makes explicit women’s initial reactions. Once the diagnosis is confirmed, a process of ‘Gaining Meaning’ commences, whereby an attempt to make sense of this ostensibly negative event begins. ‘Rebuilding’, the final stage in the process, is concerned with the extent to which women recover from the loss and resolve the inconsistency between their experience and their previous expectations.
When fetal hydronephrosis is suspected antenatally—a qualitative study Oscarsson, Gottvall & Swahnberg (2015) Sweden [19]	Hopelessness experiences of anxiety and fears about the unknown, worry and stress. Experiences of hope are based on going through crisis by knowing that you are doing the right thing, told themselves that they would deal with it after delivery and that everything would be all right at the end	To explore women’s reactions to the discovery of fetal hydronephrosis in the context of uncertainty regarding the prognosis	Qualitative study	Pregnant women	University clinic of Sweden	Ten women with antenatal diagnosis were invited to the interview 6-12 months after delivery. Mean age were 30.6 years. Six nulliparous and four parous.	The core category, ‘Going through crisis by knowing that you are doing the right thing’ illustrates the meaning of women’s reactions and feelings. It illuminates the four categories: ‘When the unexpected happens’—on the one hand, women had positive views that the suspicious malformation could be discovered; however, on the other hand, women questioned the screening. ‘To live in suspense during pregnancy’—the suspicious malformation caused anxiety and was a stressful situation. ‘Difficulties in understanding information’—the women thought they had limited knowledge and had difficulties in understanding the information. ‘Suppress feelings and hope for the best’—the women tried to postpone the problem and thought they should deal with it after delivery
Pregnancy through the Lens of Iranian Women with HIV: A Qualitative Study Zahra Behboodi-Moghadam, Khalajinia, Nasrabadi, Mohraz & Gharacheh (2015) Iran [20]	Hopelessness experiences implicit on the concerns about transmitting the virus to the baby, effects on their health. Becoming a mother after a HIV diagnosis was a source of hope, value and esteem, children seen as a “divine gift”, a chance to correct past mistakes. By participating in spiritual practices, women believed that God would respond to their needs, God would take care of their children.	To explore the experience of pregnancy among Iranian women with HIV	Qualitative study	Pregnant women	Counseling Center for Behavioral Diseases in Imam Khomeini Hospital in Tehran, Iran.	The study participants’ age ranged from 22 to 39 years. Five of them had completed primary school and 7 had finished high school. Length of time since HIV diagnosis was 1 to 5 years. A total of 7 participants had children and 5 of them reported being pregnant for the first time. Nine of the women (75%) had been infected with HIV by their husbands and 3 of them (25%) through unknown route.	Four main themes emerged from the data: fear and hope, stigma and discrimination, marital life stability, and trust in God. Despite concerns about mother-to-child transmission of HIV, and uncertain life span, HIV-infected women tended to continue their pregnancy, and having children was viewed as a window of hope for them.
Perspectives on pregnancy in women with Chronic Kidney Disease (CKD): A Semistructured Interview Study Tong, Brown, Winkelmayer, Craig & Jesudason (2015) Australia [21]	Hopelessness showed by conscious of fragility, noxious self, denied motherhood, social jealousy, fear of genetic transmission. Hope implicit on the opportunity of getting pregnant, found that the baby didn’t have the same disease, valuing life, gratitude in hindsight and focus on what is good.	To describe the beliefs, values, and experiences of pregnancy in women with CKD to inform prepregnancy counseling and pregnancy care.	Qualitative study	Pregnant women	Two renal units in Australia	41 women (95% response rate) aged 22 to 56 years with CKD stages 3 to 5 (*n* 5 5), receiving dialysis (*n* 5 5), or received a kidney transplant (*n* 5 31) from 2 renal units in Australia.	Six themes were identified: bodily failure (conscious of fragility, noxious self, critical timing, and suspended in limbo), devastating loss (denied motherhood, disempowered by medical catastrophizing, resolving grief, barriers to parenthood alternatives, and social jealousy), intransigent guilt (disappointing part- ners, fear of genetic transmission, respecting donor sacrifice, and medical judgment), rationalizing conse- quential risks (choosing survival, avoiding fetal harm, responding to family protectiveness, compromising health, decisional ownership, and unjustifiable gamble), strengthening resolve (hope and opportunity, medical assurance, resolute determination, and reticent hope), and reorientating focus (valuing life and gratitude in hindsight).
Lived experiences of women with maternal near miss: qualitative research Sabzevari, Yazdi,& Rad (2021) Iran [22]	Hopelessness implicit on fears and concerns. Unable to get pregnant again, fear of raising their child without siblings, concerns about their child without a mother, re-marriage of the spouse if the spouse wanted more children, fear of getting pregnant again and experiencing postpartum complications, failure to adjust to complications and prolonged mourning, feeling guilty, not tolerating pain, irritability, and postpartum depression. The only hope seemed to be believing in God’s will and their survival and ability to support their children.	To understand experiences and perceptions of women with higher risk pregnancy relating to problems/needs of health and care practices.	Descriptive and qualitative	Pregnant women	The interviews were conducted at the office of the educational supervisor of Sabzevar Mobini Hospital or any other place that was conveni- ent to the mothers (e.g. their home).	The mothers were selected based on their ability to express their near-miss experience and willingness to be interviewed. The age range of the selected mothers was 19–36 years.	Five main categories were extracted, including fears and concerns, failure to accept and adapt, tolerating physical and psychological pain and hardships, death experience, and medical team mismanagement. Regret and fear of raising the child with siblings, fear of the re- marriage of the spouse, and fear of complications and costs were among the subcategories of fears and concerns. Lack of adaptation to the complications and prolonged mourning were the subcategories of failure to accept and adapt, and the subcategories of tolerating physical and psychological pain and hardships were a sense of guilt, tolerating physical pain, hopelessness, irritability, hatred toward the medical team, and postpartum depression. In addition, returning to normal life, and seeing/actually feeling death were the subcategories of the death experience. The subcategories of the medical team mismanagement included medical errors, lack of sup- port/negligence,
Emotional and cognitive experiences of pregnant women following prenatal diagnosis of fetal anomalies: A qualitative study in Iran Irani, Khadivzadeh, Nekah, Ebrahimipour & Tara (2019) Iran [29]	Hopelessness is defined by disbelief, distress, panic and shock during the time of diagnosis. When their pregnancy was terminated, women experienced perinatal loss such as guilt and shame during pregnancy termination, loss of their expected child, suffering and emotional distress process, and fear of recurrence in future pregnancies. Women that decided to continue their pregnancy had a dilemma between hope and worries. In general, women carrying babies tried to keep a positive attitude towards the birth, as a way to cope with the situation. They were hopeful about the best possible outcome or return of normality. Some women hope that the problem for the health of their unborn child in the case of abnormal findings will be resolved or is miner anomaly.	To explore the emotional and cognitive experiences of pregnant women following prenatal diagnosis of fetal anomalies in Mashhad, Iran.	Qualitative study	Pregnant women	Two tertiary referral centers for fetal anomaly at Mashhad University Hospitals, Omolbanin Hospital and Imam Reza Hospital in Mashhad, Iran.	The sample studied consisted of Persian speaking parents with prenatal diagnosis of fetal anomalies at the gestational week of 12–27. All the pregnant women with a suspected or definitive diagnosis of fetal anomaly as per the ultrasound or the combined test (NT, free β-hCG and PAPP-A) were eligible for participation.	Four categories and 10 subcategories emerged. Category one, grief reactions during the time of diagnosis, contained two subcategories: shocked and panicked, and distressed and disbelieved. Category two, perinatal loss through a pregnancy termination, contained four subcategories: guilt and shame during pregnancy termination, loss of their expected child, suffering and emotional distress process, and unmet needs by health professionals. Category three, fears of recurrence in future pregnancies, had two subcategories: worried about inadequate prenatal care in the future pregnancies and worried about abnormal fetus in next pregnancies. Finally, Category four, a dilemma between hope and worries contained two subcategories: hope for normality and worried about future.
Complexity of consenting for medical termination of pregnancy: Prospective and longitudinal study in Paris Tayeh, Jouannic, Mansour, Kesrouani &Attieh (2018) Paris [30]	Hope implicit in religious beliefs and convictions and believed that their child could exceptionally survive after birth.	To analyze the patients’ perception of prenatal diagnosis of fetal cardiac pathology, and the reasons for choosing to continue with pregnancy despite being eligible to receive a medical termination of pregnancy.	Descriptive, prospective and longitudinal study	Pregnant women	Hôpital Necker—Enfants Malades in Paris, France	Eligible participants were pregnant women who decided to continue their pregnancy despite an unfavorable medical advice because they were carrying fetuses with incurable cardiac pathologies. Age between 23 and 44 years old.	Patient informed consent should be sought before any decision in neonatology, even if conflicting with the medical team’s knowledge and the pregnant mother’s benefits. Decisions to accept or decline pregnancy termination depend on the patients’ psychological character, ideologies, convictions, and mistrust in the diagnosis/prognosis, or hope in the fetus survival.
Women’s Voices: The Lived Experience of Pregnancy and Motherhood After Diagnosis With HIV Sanders (2008) New York (USA) [23]	Hopelessness characterized by periods of emotional distress, ambivalence in relation to pregnancy and mother- hood, and stigma. Hope in protecting children from contracting HIV and from HIV related stigma and hoped to rectify mistakes made with children born previously and to be a good, loving mother.	The study aimed to explore the meaning of pregnancy after diagnosis with HIV	Qualitative study	Pregnant women	Interviews conducted in two academic health centers in metropolitan New York.	Participants were a purposive sampling of 9 women, 34 to 53 years old, who had been diagnosed with HIV and were currently pregnant or who had become mothers postdiagnosis.	The result of the study included themes of extreme emotional distress after HIV diagnosis, feeling stigmatized, emotions related to the pregnancy and baby, experiences with health care providers, and as positive and supporting.
The lived experiences of rural women diagnosed with the human Immunodeficiency virus in the antenatal period Fords, Crowley & Merwe (2017) South Africa [25]	Hopelessness implicit in a negative self-image, loneliness, feelings of isolation, fear from loved ones, women experienced blame, fear, the cruelties of stigma, stereotyping and judging an avoided any romantic relationship. Fear of being ill, being in a hospital or dying. Women felt hope to live and see the future of their children, hope on their spiritual beliefs. The initiation of ART gave them hope as they were confident that the treatment would improve their health, extend their life, protect their unborn children and even cure HIV. The most important hope that they had for the future was that their unborn child would be HIV free	To explore the lived experiences of women diagnosed with HIV in the antenatal period in a rural area in the Eastern Cape province of South Africa.	Phenomenological study	Pregnant women	Pregnant women residing in the Maluti local service area in the Eastern Cape who attended one the local clinics.	Ten women over the age of 18, diagnosed with HIV for the first time in the antenatal period of pregnancy.	Women diagnosed with HIV during pregnancy are ultimately concerned with the wellbeing of their unborn children, and this concern motivates their adherence to ART. Women’s lived experiences are situated in their unique sociocultural context, and although some known challenges remain, counselling and support strategies need to be informed by exploring context-specific issues and involving the local community.
The Lived Experiences of HIV-Positive, Pregnant Women in Thailand Ross, Sawatphanit, Wilaiphan; Burke & Suwansujarid (2007) Thailand [24]	Hopelessness: Women perceived their lives as a struggle. Struggling alone and experiences of shock, fear, anxiety and depression; Sharing one’s struggling, with fears of stigmatization and discrimination; struggling for the baby, most postpartum Thai mothers indicated that their babies were central to their determination in helping them to move on with their lives; Struggling through ups and downs Hope: Women found hope through the taking of antiretroviral medicines and, subsequently, showed a desire to fight the virus as long as possible for their children. For health care professionals, when a seropositive pregnant woman who has her baby as her hope is feeling “down,” gently reminding her of her unborn baby could be a help in lifting her hope and spirit	The purpose of our study was to examine the lived experiences of 10 pregnant women in Thailand following their HIV diagnosis	Phenomenological study	Pregnant women newly diagnose with HIV	Prenatal clinic at a government hospital in Thailand	All participants were Buddhist. They ranged in age from 18 to 29 years. Five were graduates of primary school, 3 had completed junior high school, and 2 had finished high school. Nine had a monthly low family income. Only one participant had a middle-class family. Eight were married or lived with a partner, and 2 were divorced.	All participants in these study decided not to end their pregnancies. This might be explained in the fact that all women were Buddhist, with Buddhists usually believing that terminating one’s life or ending a pregnancy is a big sin. From a health care perspective, identifying helpful resources such as a peer/support group can be critical for a woman when she is ready to share her struggle with others. Peer support was found to be helpful for 7 women in our study and has been found to be effective across cultures and countries in reducing seropositive women’s fears, depression, and attempts at suicide. In general, support from health care professionals to assist the HIV-positive mother’s efforts to promote her baby’s health will be of great value, regardless of the ultimate diagnosis of the baby’s HIV status.
The experiences of women with maternal near miss and their perception of quality of care in Kelantan, Malaysia: a qualitative study Norhayati, Hazlina, Nik Hussain; Asrenee & Sulaiman (2017) Malaysia [26]	Hopelessness: several forms of negative emotions such as fear, anxiety, alarm, incomplete self, discouragement and numbness. Fear on their own lives and lives of their babies, fear for the prenatal outcomes and of undergoing surgery, fear of recurrence similar incidents and inability to conceive in the future, sense of death; incomplete self, because being a woman without a uterus and without a baby; sadness for not being able to have more children Hope: The women adapted to most of negative emotions and to difficult life events, like traumatic childbirth by anchoring their reasoning to religiosity and faith and appeared to have accepted the situation calmly. They responded to their situations positively, delegating the resolution to God and regarding what had happened as what God had planned for them. They were very grateful that God gave them a second chance. Other forms of adaptation included natural maternal disposition in which their attention were distracted and focused on their children and that was a motivator to seek treatment and as a source of strength to continue living. The competency of the healthcare providers in the form of adequate knowledge and skills in providing optimal care had gained trust from the women. Emotion and social support and improved relationship quality were associated with better mental health and well-being, reduced stress and protection from postpartum depression. In the current study, social support appeared to play a role in protecting the women from ill health. Despite their experiences, the women were relieved at having survived their acute, severe complications and looked forward to resuming their lives normally.	This study aimed to explore the experiences of women with maternal near miss and their perception of the quality of care in Kelantan, Malaysia	Qualitative phenomenological approach	All women screened for the presence of any vital organ dysfunction or failure based on the World Health Organization criteria for maternal near miss.	All women admitted to labour room, obstetrics and gynaecology wards and intensive care units in 2014	Thirty women who had experienced maternal near miss events were included in the analysis. All were Malays between the ages of 22 and 45. Almost all women (93.3%) had secondary and tertiary education and 63.3% were employed.	In appraising the maternal near miss events, the study found that the women viewed their experiences as frightening and that they experienced other negative emotions and a sense of imminent death. Their perceptions of the quality of their care were influenced by the competency and promptness in the provision of care, interpersonal communication, information-sharing and the quality of physical resources. These factors should be of concern to those seeking to improve services at healthcare facilities. The predisposition to seek healthcare was influenced by costs, self-attitude and beliefs.
The lived experience of pregnancy while carrying a child with a known, nonlethal congenital abnormality Hedrick (2004) USA [32]	Hopelessness is define by the loss of the perfect baby with feelings of grief, shock, anger and guilt. Hope defined with good sources of information, time to prepare, support from family and friends, spiritual beliefs and staying busy with work and other activities and empathizing with the baby	To gain an understanding of the experience of pregnancy while carrying a child with a known, nonlethal congenital abnormality	Phenomenological study	Pregnant women	Outpatient perinatal center at a large Midwestern hospital	Ages between 18 and 44 years. Gestacional age at the time of the diagnosis 17 to 26 weeks. Interviewed between 24 and 36 weeks. Fetal diagnosis of neural tube defect, cleft lip, congenital heart defect, renal anomaly, cystic malformation of the long and down syndrome	The pregnancy experience was of a paradoxical nature. Knowledge of fetal diagnosis with positive and negative consequences. Time is good but also the enemy; you grieve but you do not grieve; my baby’s not perfect, but he’s still mine
Feelings and expectations of pregnant women living with HIV: A phenomenological study Arcoverde, Conter, Silva & Santos (2015) Brazil [27]	Hopelessness: - Human suffering and anxiety resulting from stigma, prejudice and discrimination - fear of exposure, prejudice and discrimination produced by the stigma Hope: - Motherhood has such a strong meaning to these women that not even the possibility of transmitting the virus to the foetus can change their mind. The wish to be a mother is stronger than the problems faced throughout life. - Mothers with HIV focus their life on the uninfected child, which symbolizes the continuity and the hope to overcome their fears. The child can be the motivator that helps them to face the challenges imposed by the disease. - For them, positive pregnancy experiences were an important source of support and hope to carry on living and taking care of their own health. The participants accepted their pregnancy and asked for a healthy baby. Even if it is an unplanned pregnancy, the child becomes a motivating force, giving them reason to fight the disease. - A mother living with HIV will adhere more easily to treatment if she has been informed about the seriousness of the disease and about the possibilities of the child being infected. - Pregnant women make adjustments to deal with their seropositivity through religion, seeking in God’s figure love, care, help, strength, forgiveness, and well-being. The support of religion brings hope conveying a feeling of comfort and has an impact on coping with HIV/AIDS - For these women, the unborn child is the motivation for them to rethink their life and to withstand the bad times. Religiousness resurfaced by the wish for a child gives meaning to their life and helps the construction of a female identity - Faith is a life support that helps these women to withstand the uncertainties of being pregnant and HIV positive. Their faith in God gave them the confidence to face the difficulties imposed by the disease and hope for better days for them and their children - Spirituality is a comfort that helps them to bear the pain of being HIV positive; it makes them believe in quality of life due to treatment and, in the hope of a miracle, that God may transform their lives completely	The objective of this research was to identify the feelings and expectations of such pregnant women about the disease and pregnancy	Phenomenological qualitative research based on Maurice Merleau-Ponty’s philosophy of perception	Pregnant women with HIV diagnosis	Outpatient unit of the SAE of Foz do Iguaçu, in the state of Parana	Five pregnant women diagnosed with HIV monitored in SAE of Foz do Iguaçu, in the state of Paraná, participated in the study. They were married, aged between 20 and 35 years old, and had been diagnosed from one month to ten years before research began	The interviews conveyed the experiences of the women with HIV, their acceptance of the limitations imposed by the disease and showed how they dealt with the stigma surrounding HIV. Despite the prejudice, such pregnant women did not lose faith and hope. Pregnant women believe in the treatment and the possibility of their children being born healthy. The desire of motherhood increases their expectations about the care, which prevents complications from the infection. The study participants accepted the pregnancy, mainly because the desire to become a mother was stronger than anything else they could feel. The treatment was then accepted, as the only way to protect their children from a HIV infection. The researchers identified feelings of strength, will, and determination to overcome the problems which transcend the difficulties encountered throughout pregnancy. The fear of harming the child—symbol of perseverance, wishes and hopes—is faced and reignites their desire to carry on living in order to care for their children and to protect them. Communication and education for the construction of new concepts and ideas related to the phenomenon can indicate new ways to learn about change processes. Social movements, cultural changes, as well as social equality and inequality; action and intervention based on fair policies can also be one of the responses to the problem.
‘We did everything we could’—a qualitative study exploring the acceptability of maternal-fetal surgery for spina bifida to parents Crombag, Sacco, Stocks, Vloo, Merwe, Gallagher, David, Marlow & Deprest (2021) Belgium and United Kingdom [31]	Hopelessness: - Uncertainty of impact of the surgery on their future child’s quality of life. - Fear. Potential complications of the surgery - Fear of losing their unborn child and fear of not waking up after the operation - Post-traumatic stress and depression Hope: - Strong feelings of responsibility and determination to do anything to improve their future child’s health outcomes - Only option to improve their child’s outcome - an opportunity they were given - MFS provided hope for their child’s future	To explore the concepts and strategies parents employ when considering maternal-fetal surgery (MFS) as an option for the management of spina bifida (SB) in their fetus, and how this determines the acceptability of the intervention	Qualitative study	Parents	Two MFS partner centres with specialist assessment (University Hospitals Leuven, Belgium; University College London Hospital, United Kingdom)	Parents whose fetuses with SB were eligible for MFS, Age above 18 years old.	MFS for SB remains highly acceptable from diagnosis until 3–6 months postnatally. For those opting for MFS, expectations seemed to be realistic yet were driven by hope and expectation of the best outcome. For parents opting for termination of pregnancy, the potential benefit of MFS seems to play a minimal role in their final decision

## Appendix C

**Table:**

**Article**	**Abstract and Title**	**Introduction and Aims**	**Method and Data**	**Sampling**	**Data Analysis**	**Ethics and Bias**	**Results**	**Transferability or Generalizability**	**Implications and Usefulness**	**TOTAL**
Badakhsh, Hastings-Tolsma, Firouzkohi, Amirshahi & Hashemi (2020) [18]	4	4	4	4	4	4	4	4	4	36
Lalor, Begley & Galavan (2009) [28]	4	4	4	4	4	4	4	4	4	36
Oscarsson, Gottvall & Swahnberg (2015) [19]	4	4	4	4	4	4	4	4	4	36
Behboodi-Moghadam, Khalajinia, Nasrabadi, Mohraz & Gharacheh (2015) [20]	4	4	4	4	4	4	4	3	3	34
Tong, Brown, Winkelmayer, Craig & Jesudason (2015) [21]	4	4	4	4	4	4	4	4	4	36
Sabzevari, Yazdi, & Rad (2021) [22]	4	4	4	4	4	3	4	3	3	33
Irani, Khadivzadeh, Nekah, Ebrahimipour & Tara (2019) [29]	4	4	4	4	4	4	4	4	4	36
Tayeh, Jouannic, Mansour, Kesrouani &Attieh (2018) [30]	4	4	4	4	4	3	4	4	4	35
Sanders (2008) [23]	3	4	4	4	4	4	4	4	4	35
Fords, Crowley & Merwe (2017) [25]	4	4	4	4	4	4	4	4	4	36
Ross, Sawatphanit, Wilaiphan; Burke & Suwansujarid (2007) [24]	4	4	4	3	4	4	4	3	3	33
Norhayati, Hazlina, Nik Hussain; Asrenee & Sulaiman (2017) [26]	4	4	3	4	3	3	4	3	4	32
Hedrick (2004) [32]	4	4	3	4	3	3	4	4	3	32
Arcoverde, Conter, Silva & Santos (2015) [27]	4	4	4	4	3	4	3	3	3	32
Crombag, Sacco, Stocks, Vloo, Merwe, Gallagher, David, Marlow & Deprest (2021) [31]	4	4	4	4	4	4	4	4	4	36
